# Caries experience, oral health behaviors, and socioeconomic determinants among high school students in Madinah, Saudi Arabia: a cross-sectional study

**DOI:** 10.3389/froh.2026.1850464

**Published:** 2026-09-08

**Authors:** Ahmed A. Marghalani, Zaina E. Kalthoum, Shadan H. Sharbib, Jawan F. Alyenbaawi, Taif S. Alahmadi, Zainab A. Alghamdi, Lama M. Alkahtani, Faten S. Alharbi, Shatha A. Alhujaily, Faisal F. Hakeem

**Affiliations:** 1 Department of Preventive Dental Sciences, Dental College and Hospital, Taibah University, Madinah, Saudi Arabia; 2 Dental College and Hospital, Taibah University, Madinah, Saudi Arabia

**Keywords:** caries experience, DMFT, Madina, oral health behaviour, Saudi arabia

## Abstract

**Background:**

Dental caries remains a prevalent global health concern, affecting millions despite being preventable. In Saudi Arabia, especially in Madinah, data on caries prevalence among adolescents is limited. This gap is addressed via assessing caries prevalence and associated risk factors.

**Methodology:**

This cross-sectional study enrolled 2,514 high school students from 24 schools in Madinah, selected through stratified multistage cluster sampling. Data were collected through a self-administered questionnaire and clinical examinations using DMFT index. Chi-square tests and logistic regression were used.

**Results:**

The overall prevalence of dental caries was 81.5%, with a mean DMFT score of 4.07 (SD = 3.64). Students attending public schools (87.3%) and those with lower parental education were more likely to have high/very high CE (*p* < 0.001). Self-rated oral health was significantly associated with CE, with students reporting better oral health having lower caries experience (*p* = 0.02). Logistic regression analysis revealed that being female (OR = 1.32, *p* = 0.004), increasing age (OR = 1.14, *p* = 0.002).

**Conclusion:**

Dental caries is highly prevalent among high school students, with significant associations between caries experience and gender, and oral health behaviours, which highlight the need for targeted oral health promotion and prevention strategies.

## 1 Introduction

Dental caries, commonly known as tooth decay, is a prevalent chronic infectious disease resulting from tooth-adherent cariogenic bacteria that metabolize sugars to produce acid, which over time demineralizes tooth (1). Despite being preventable, the widespread prevalence of dental caries continues to affect a significant number of individuals worldwide, posing a substantial burden on public health systems (2). Kassebaum et al., 2017 investigated the prevalence of untreated dental caries on global and regional levels worldwide (1990–2015) and highlighted that the prevalence of untreated caries in permanent teeth was estimated to be between 2.4 to 2.7 billion individuals (2).

In Saudi Arabia, dental caries is a significant oral health concern. A systematic review conducted by Alshammari et al., (2021) (3) examined the prevalence of dental caries in both primary and permanent dentition in different regoins of Saudi Arabia (7 out of 13 regoins, including Riyadh, the Eastern Province, and the Western region) from 1999 to 2019. They included 49 studies, which yield a wide range of caries proportions, ranging from 0.21 to 1.00 for primary teeth and 0.05–0.99 for permanent teeth. The study concluded that the methodological quality of the included studies was poor. It is important to note that the available data on a national level of dental caries prevalence in Saudi Arabia is limited, and the current studies do not provide a comprehensive assessment of the extent of dental caries across the country. There remains a significant gap in the national-level data on dental caries prevalence, especially in adolescents. Data specific to Madinah city are particularly scarce, with most of the available studies in this region focusing on younger school-age children rather than high school adolescents.

Most existing research in Saudi Arabia has focused on younger children aged 6–15 years (4–8) while other age groups remain understudied. Specifically, there is a lack of research investigating the prevalence of dental caries among high school students, particularly in Al-Madina region. Therefore, the aim of this study is to address the existing knowledge gap by assessing the prevalence of dental caries and investigate the associations between caries experience, oral health behaviors, and socioeconomic factors among high school students in Madinah, Saudi Arabia.

## 2 Methods

### 2.1 Study design and sample selection

This study employed a cross-sectional design using a stratified multistage cluster random sampling method. Initially, a comprehensive list of high schools in Madinah city was obtained. The sampling process followed the Ministry of Education's administrative divisions, which consist of four regions: northern, eastern, western, and southern. Schools were randomly selected from each of these regions, and within each selected school, random classes were chosen to participate. Both government and private schools were included in the sampling frame. The randomization process ensured a nearly equal selection of male and female students, as well as a representative distribution across different socioeconomic groups.

### 2.2 Sample size calculation

The sample size was calculated using population data from the Saudi Authority of Statistics, which reported 23,719 male and 26,169 female high school students in Madinah. Assuming a 95% confidence level, 5% margin of error, and caries prevalence of 65%, the minimum required sample size after finite population correction was 345 students per gender group. The final analytical sample included 2,514 students, comprising 1,249 males and 1,265 females.

### 2.3 Study population

The target population consisted of 1st-year high school students (chronological age 15–16 years) in Madinah, Saudi Arabia, enrolled and actively attending school at the time of examination. This age group was selected in accordance with WHO oral health survey guidelines, as all permanent teeth (excluding third molars) have typically erupted by this stage and have been exposed to the oral environment for three to nine years, making this population ideal for assessing caries prevalence.

Inclusion criteria required active school enrollment and willingness to participate in the study. Exclusion criteria included students with mental or psychological conditions that prevented them from accurately interpreting the questionnaire, and those whose parents did not provide written informed consent. Only students meeting all inclusion criteria and providing assent were enrolled.

### 2.4 Clinical examination

Examiners were trained over a two-day period to standardize caries assessment and ensure calibration. Clinical examinations were conducted by trained and calibrated 6th-year dental students to assess the prevalence of caries and calculate DMFT (Decayed, Missing, Filled Teeth) scores, using a standardized coding system developed by the World Health Organization (WHO). The system employs numerical codes from 0 to 9 to diagnose and record the status of permanent teeth. Examinations were conducted in schools' venue, each equipped with two separate, isolated examination chairs to ensure participant privacy. The process began with an explanation of the examination procedure. Clinical examinations were performed using an artificial light.

### 2.5 Questionnaire

A self-administered, questionnaire adapted from the WHO Oral Health Questionnaire for Children and Adults (2013) was used to collect information on various aspects of oral health. The questionnaire gathered demographic data, including sex, age, current educational level, and living location (city or rural area). It also inquired about parental occupation and education, with options ranging from “illiterate” to “university degree or higher.” Participants were asked to rate their oral health as “excellent,” “very good,” “good,” “fair,” or “poor.” Additionally, Participants were asked about the frequency of dental visits in the past year, with options such as “once,” “twice,” “three times or more,” “not once during the last year,” and “never been to the dentist.” To assess oral hygiene habits, participants were asked about their toothbrushing frequency, with options such as “I do not brush my teeth,” “2–3 times a month,” “once a week,” “2–6 times a week,” “once a day,” or “twice or more a day.”.

### 2.6 Statistical analysis

The data analysis was conducted using Stata 18 software. The primary outcome variable, caries experience, was measured using the Decayed, Missing, Filled Teeth (DMFT) index, which was categorized into low/moderate (0–5) and high/very high (6 and above). A binary variable for caries experience (CE) was created to indicate low/moderate vs. high/very high caries experience. The Care Index (CI), which reflects the proportion of filled teeth relative to total DMFT, was calculated using the formula (Filled Teeth/DMFT) * 100 to measure the level of restorative care. Independent variables included demographic factors (sex, age, year of study), socioeconomic factors (father's and mother's education levels, school type), and oral health behaviors (self-rated oral health, brushing frequency, and dental visits). Descriptive statistics, including means and standard deviations for continuous variables and frequencies and percentages for categorical variables, were generated to summarize the characteristics of the study population.

The distribution of DMFT-related variables was assessed using graphical methods and normality testing. As these variables were not normally distributed, Mann—Whitney U and Kruskal—Wallis tests were used for continuous variables, as appropriate, while Chi-square tests were used for categorical variables. Multivariable logistic regression was used to evaluate the association between caries experience and potential risk factors, adjusting for relevant demographic, socioeconomic, and oral health-related variables. Odds ratios (ORs) with 95% confidence intervals (CIs) were reported, and statistical significance was set at *p* < 0.05.

### 2.7 Ethical considerations

Ethical approval was granted from the Research Ethical Committee at Taibah University (TUCDREC/280l24/FHakeem)**.** Informed consent was obtained from each participant individually, accompanied by an explanation of the process to ensure their understanding. The study followed guidelines recognized by the Declaration of Helsinki.

## 3 Results

The total study sample consisted of 2,514 high school students from Madinah, Saudi Arabia, with a nearly equal distribution between males and females. The mean age of the participants was 16.4 years (SD = 0.9), with most students being in their first year of high school (59%). A significant portion of the students' parents had either intermediate (39.9%, *n* = 1,000) or high levels of education (45.1% for mothers; 48.2%for fathers). Most students attended public schools (83.1%) with a smaller proportion attending private schools (16.9%).

The mean DMFT score of the sample was 4.07 (SD = 3.64). Caries prevalence was high, with 81.5% of participants exhibiting at least one decayed, missing, or filled tooth, while 18.5% had no caries experience. Regarding caries severity, 25.46% of participants were classified as having a very low DMFT score, 12.57% were in the low category, and 25.42% had moderate scores. A significant portion of the sample fell into the higher categories, with 15.95% categorized as high and 20.6% as very high. In a binary categorization of DMFT, 36.56% of participants had high or very high DMFT scores, while 63.44% had low to moderate DMFT scores.

 Table 1 shows the comparison of demographic and socioeconomic factors between participants with low/moderate CE and those with high/very high CE. A significant difference in caries experience was observed between males and females, with a higher proportion of females in the high/very high CE group (57.3% vs. 42.7%, *p* < 0.001). There was no significant difference in age between the groups (*p* = 0.07). Year of study was significantly associated with CE (*p* < 0.001), with a higher percentage of participants in the third grade having high/very high CE compared to first- and second-grade students. Additionally, parental education showed significant associations with CE. Mothers with higher education levels were more likely to have children with low/moderate CE (*p* < 0.001), while fathers' education also showed a significant association (*p* = 0.018). Participants attending public schools had a higher likelihood of having high/very high CE compared to those attending private schools (87.3% vs. 80.7%, *p* < 0.001).

**Table 1:** Comparison of demographic and socioeconomic factors by caries experience (low/moderate vs. high/very high) among study participants (*N* = 2,514).

Variable	Group	Low/Moderate CE (*n* = 1,595)	High/very high CE (*n* = 919)	Total (*n* = 2,514)	*P*-value
Sex	Male	857 (53.7)	392 (42.7)	1,249 (49.7)	<0.001
	Female	783 (46.3)	527 (57.3)	1,256 (50.3)	
Age, mean (SD)		16.3 (0.9)	16.5 (1.1)	16.4 (0.9)	0.07
Year of Study	First grade	983 (61.6)	500 (54.4)	1,483 (59)	<0.001
	Second grade	369 (23.1)	242 (26.3)	611 (24.3)	
	Third grade	243 (15.2)	177 (19.3)	420 (16.7)	
Mother Education	Low	211 (13.3)	164 (17.9)	375 (15)	<0.001
	Intermediate	624 (39.3)	367 (41.1)	1,000 (39.9)	
	High	754 (47.5)	375 (41)	1,129 (45.1)	
Father Education	Low	138 (8.7)	103 (11.3)	241 (8.7)	0.018
	Intermediate	655 (41.2)	400 (43.8)	1,055 (42.2)	
	High	795 (50.1)	411 (45)	1,206 (48.2)	
School type	Public	1,278 (80.7)	802 (87.3)	2,089 (83.1)	<0.001
	Private	308 (19.3)	117 (12.7)	425 (16.9)	

 Table 2 presents the comparison of oral health status, dental visit frequency, and brushing habits between males and females. Females had significantly higher mean DMFT scores compared to males (4.5 vs. 3.6, *p* < 0.001). The Care Index, which measures the proportion of filled teeth relative to the total DMFT, was significantly higher among females (4.25%) compared to males (2.89%, *p* = 0.01). Self-rated oral health also differed significantly by sex, with 88.2% of females reporting their oral health as good, very good, or excellent, compared to 82.2% of males (*p* < 0.001). Dental visit frequency showed significant differences, with females reporting more frequent dental visits. For instance, 39.4% of females had three or more dental visits in the past year, compared to 19.3% of males (*p* < 0.001). Brushing habits also varied significantly by sex. A higher proportion of females reported regular brushing (88.5%) compared to males (56%, *p* < 0.001), while males had higher frequencies of rare or irregular brushing.

**Table 2:** Summary of oral health status, dental visits, and brushing habits by sex for study participants (*N* = 2,514).

Variable	Categories	Male (*n* = 1,249)	Female (*n* = 1,256)	Total (*n* = 2,514)	*P*-value
D, mean (SD)		3.2 (2.9)	3.6 (3.1)	3.41 (3)	0.001
				
M, mean (SD)		0.3 (0.8)	0.7 (1.4)	0.5 (1.2)	<0.001
				
F, mean (SD)		0.1 (0.4)	0.2 (0.5)	0.1 (0.5)	<0.001
					<0.001
DMFT, mean (SD)		3.6 (3.3)	4.5 (3.8)	4.1 (3.6)	<0.001
				
Care Index %		2.89%	4.25%	3.57%	0.001
				
Self-rated oral health	Good/V.good/Excellent	1,026 (82.2)	1,114 (88.2)	2,140 (85.2)	<0.001
	Fair/poor	222 (17.8)	149 (11.8)	371 (14.8)	
Dental visits frequency during last year	Never received dental care	335 (26.9)	164 (13.0)	499 (19.9)	<0.001
	once	157 (12.6)	139 (11.0)	296 (11.8)	
	Two times	191 (15.3)	226 (17.9)	417 (16.6)	
	Three times or more	240 (19.3)	497 (39.4)	737 (29.4)	
	Did not visit during last year	322 (25.9)	235 (18.6)	557 (22.2)	
Brushing frequency	Never	85 (6.8)	12 (1.0)	97 (3.9)	<0.001
	Rare	285 (22.9)	57 (4.5)	342 (13.6)	
	Irregular brushing	178 (14.3)	76 (6.0)	254 (10.1)	
	Regular brushing	697 (56.0)	1,117 (88.5)	1,814 (72.4)	

Continuous variables are presented as mean (SD); between-sex comparisons were performed using the Mann—Whitney U test due to non-normal distribution. Categorical variables are presented as *n* (%) and compared using the Chi-square test. Results in this table are unadjusted bivariate comparisons.

 Table 3 presents the results of the logistic regression analysis exploring factors associated with high/very high caries experience. Age was significantly associated with caries experience, with an increase in age corresponding to higher odds of having high/very high CE (OR = 1.14, 95% CI: 1.05–1.25, *p* = 0.002). Females had 1.32 times higher odds of having high/very high CE compared to males (*p* = 0.004). School type was also a significant factor, with participants attending private schools having lower odds of high/very high CE (OR = 0.71, 95% CI: 0.55–0.93, *p* = 0.012) compared to those attending public schools. Mother's education showed a protective effect, with participants whose mothers had high education levels having significantly lower odds of high/very high CE (OR = 0.67, 95% CI: 0.50–0.91, *p* = 0.009). The association for father's education was not significant. Frequent dental visits were strongly associated with higher odds of high/very high CE. Participants who visited the dentist twice (OR = 2.09, *p* < 0.001), three or more times (OR = 2.41, *p* < 0.001), or did not visit during the last year (OR = 1.53, *p* = 0.003) had significantly higher odds of having high/very high CE compared to those who never received dental care. Brushing frequency was not significantly associated with caries experience, although irregular and regular brushing both showed lower odds compared to those who never brushed, but the results were not statistically significant.

**Table 3:** Logistic regression showing the association between caries experience and potential risk factors among high school students in Madinah, Saudi Arabia (*n* = 2,514).

Variables	Caries Experience	OR	[95% Conf	Interval]	*p*-value	Sig
Age in years		1.14	1.05	1.25	.002	***
Sex	Male	(Reference)				
	Female	1.32	1.09	1.60	.004	***
School type	Governmental	(Reference)				
	private	0.71	0.55	0.93	.012	**
Father Education	Low	(Reference)				
	Medium	0.86	0.62	1.18	.367	
	High	0.81	0.58	1.14	.235	
Mother Education	Low	(Reference)				
	Medium	0.77	0.58	1.01	.063	*
	High	0.67	0.50	0.91	.009	***
Brushing frequency	Never	(Reference)				
	Rare	0.77	0.47	1.25	.304	
	Irregular	0.83	0.50	1.38	.489	
	Regular	0.82	0.52	1.28	.39	
Dental visit frequency	Never received dental care	(Reference)				
	Once	1.83	1.33	2.51	.01	***
	Twice	2.09	1.56	2.8	.01	***
	Three or more	2.41	1.85	3.14	.01	***
	Did not visit	1.53	1.16	2.02	.003	***

OR, odds ratio; CI, confidence interval. Adjusted ORs were estimated using multivariable logistic regression including age, sex, school type, father's education, mother's education, brushing frequency, and dental visit frequency. No stepwise or backward selection was applied.

*** *p* < 0.01.

** *p* < 0.05.

* *p* < 0.10.

## 4 Discussion

In this study, the prevalence of dental caries was 81.5% among the adolescents of Madinah. Two studies were done in Saudi Arabia where they reported lower percentages of dental caries prevalence and they were Orfali et al. (7) (61.7%), and a systematic review by Al Agili (9) (70%). However, it is worth noting that other studies included more age groups than the age groups included in the present study. Notably, the prevalence in Madinah (81.5%) is substantially higher than reports from Riyadh and Qassim regions, suggesting potential regional disparities. These variations warrant investigation into local factors including urban versus rural distribution, socioeconomic gradients, regional dietary habits (particularly local consumption of sweetened beverages and confectioneries), variations in water fluoridation status across provinces, and differential implementation of public health prevention programs within Saudi Arabia.

On the other hand, higher prevalence of dental caries has been highlighted among females than males (*P* = 0.004), which can be attributed to several factors. While the pattern of eruption of female teeth begins earlier than that of male teeth, and that means that female teeth are exposed to the oral environment, bacteria, and their substrates for longer periods of time than the teeth of a male of the same age, this biological factor alone is insufficient to explain such a pronounced gender difference in a high school cohort. Multiple additional factors likely contribute to this observed disparity. Behavioral differences during adolescence, including distinct dietary preferences such as increased frequency of snacking and consumption of sugary beverages, are more prevalent among females in many populations. Furthermore, psychological stress during adolescence may differentially affect salivary flow rates and buffering capacity between genders, thereby influencing caries susceptibility. Regional and cultural factors specific to Madinah may also play a role, including gender-specific dietary practices or differential healthcare-seeking behaviors. These multifactorial contributors should be considered when interpreting the gender-based differences observed in this study. The finding of this study contrasts with the results of a previous study conducted by Andegiorgish et al. (10), which found no significant association between sex and caries prevalence although the sample of Andegiorgish et al. study included only 12 years old children, suggesting that gender-based behavioral and physiological differences may become more pronounced during late adolescence.

The mean DMFT reported among the participants of this study is 3.8 (± 3.2). Several studies reported a comparable mean DMFT of 3.5 (8, 9, 11). However, AlDosari et al. (12) reported a higher mean DMFT of 6.53 among primary school children in both Riyadh and Qassim. The differences in the mean could potentially be attributed to variations in the age groups of the studies and differential treatment patterns across regions, highlighting the need for region-specific prevention strategies.

Differences in parental SES were found to have a significant association with the caries experience of the participants, where participants with higher levels of parental education had lower caries experience. This finding aligns with the results reported in previous studies (13, 14). In addition, the type of school attended, where a study done by Vasireddy et al. highlighted a significantly more association with caries experience among students in public schools compared to those studying in private schools. These findings underscore the influence of socioeconomic factors on oral health outcomes and suggest that targeted prevention programs should prioritize students from lower SES backgrounds.

One significant finding that has been reported by this study is the presence of an association between using charcoal and caries experience (*P* = 0.019). While the number of participants who reported using charcoal (*n* = 146) was significantly lower than those who do not use it (*n* = 1,052), this statistically significant finding warrants substantive discussion rather than dismissal. Charcoal products are highly abrasive and promote increased surface roughness of dental enamel, thereby facilitating plaque retention and bacterial colonization. Furthermore, most charcoal-based oral products lack active fluoride components and evidence-based anti-caries properties, distinguishing them from fluoridated conventional toothpastes. The abrasive nature of charcoal, combined with the absence of protective agents, likely explains the observed association with increased caries experience. This finding has important public health implications, particularly in regions where charcoal-based oral products remain popular traditional.

On the other hand, the study reported no significant association between several factors. Firstly, the frequency of tooth brushing and caries experience, since the participants who reported no brushing at all exhibited a lower caries experience than expected. This paradoxical result is unlikely to reflect a true biological effect and is more likely explained by limitations of the self-reported questionnaire data. Adolescent participants may have over-reported favorable brushing habits due to social desirability bias, feeling reluctant to admit poor oral hygiene in a school setting, or the discrepancy may simply reflect inattentive or low-engagement responding given the length of the self-administered questionnaire. We consider this a major limitation of the self-reported component of the present study, separate from the clinically examined DMFT outcome, and recommend that future studies validate brushing frequency through objective or observational measures where possible. Secondly, the present study revealed no significant association between fluoride knowledge and using Miswak and caries experience, where those who lacked knowledge about fluoride or did not use miswak had a low caries experience. The findings of previous studies contradict our results (15, 16), which may again be related to the self-reported nature of these variables in our questionnaire.

## 5 Limitations

This study has several limitations that should be considered when interpreting the findings. First, the cross-sectional design does not allow for causal inference between the identified risk factors and caries experience, and the observed associations should therefore be interpreted as correlational rather than causal. Second, behavioral data, including brushing frequency, dental visit history, and use of products such as charcoal and Miswak, were collected through a self-administered questionnaire and are therefore subject to recall and social desirability bias, as reflected in the counterintuitive brushing findings discussed above. Finally, although schools were selected from across the four administrative regions of Madinah using a stratified multistage cluster sampling method, the study was confined to a single city, which may limit the generalizability of the findings to other regions of Saudi Arabia that differ in socioeconomic and environmental profile. Future longitudinal studies incorporating objective measures of oral hygiene behavior and multi-regional sampling are recommended to build on these findings.

## 6 Conclusion

This study highlights the high prevalence of dental caries (81.5%) among adolescents in Al-Madinah, Saudi Arabia, indicating the need for proactive measures to address this public health concern. Considering these findings, we strongly recommend the development and implementation of oral health prevention programs targeted at this age group. These programs should aim to raise awareness about dental caries prevention, promote good oral hygiene practices, and emphasize the importance of regular dental check-ups. Additionally, it is crucial to expand dental health services to ensure adequate access and provision of care for adolescents to effectively address their oral health needs.

## Data Availability

The raw data supporting the conclusions of this article will be made available by the authors, without undue reservation.

## References

[1] Rathee M Sapra A. Dental caries. InStatPearls [Internet]. Treasure Island, FL: StatPearls Publishing (2023).

[2] Kassebaum NJ Smith AGC Bernabé E Fleming TD Reynolds AE Vos T. Global, regional, and national prevalence, incidence, and disability-adjusted life years for oral conditions for 195 countries, 1990–2015: a systematic analysis for the global burden of diseases, injuries, and risk factors. J Dent Res. (2017) 96:380–7. 10.1177/0022034517693566 28792274 PMC5912207

[3] Alshammari FR Alamri H Aljohani M Sabbah W O'Malley L Glenny AM. Dental caries in Saudi Arabia: a systematic review. J Taibah Univ Med Sci. (2021) 16:643–56. 10.1016/j.jtumed.2021.06.008 34690643 PMC8498786

[4] Adam TR Al-Sharif AI Tonouhewa A AlKheraif AA. Prevalence of caries among school children in Saudi Arabia: a meta-analysis. Adv Prev Med. (2022) 2022:7132681. 10.1155/2022/7132681 36105432 PMC9467753

[5] Aqeeli A Alsharif AT Kruger E Tennant M Bakeer H. Caries prevalence and severity in association with sociodemographic characteristics of 9–12-year-old school children in Al-Madinah, Saudi Arabia. Saudi Dent J. (2021) 33:897–903. 10.1016/j.sdentj.2021.09.008 34938031 PMC8665159

[6] Farooqi FA Khabeer A Moheet IA Khan SQ Farooq I ArRejaie AS. Prevalence of dental caries in primary and permanent teeth and its relation with tooth brushing habits among schoolchildren in eastern Saudi Arabia. Saudi Med J. (2015) 36:737–42. 10.15537/smj.2015.6.10888 25987118 PMC4454910

[7] Orfali SM Alrumikhan AS Assal NA Alrusayes AM Natto ZS. Prevalence and severity of dental caries in school children in Saudi Arabia: a nationwide cross-sectional study. Saudi Dent J. (2023) 35:969–74. 10.1016/j.sdentj.2023.09.008 38107051 PMC10724354

[8] souror Y. Dental caries prevalence status in Saudi Arabia and neighboring eastern countries in the last decade review. Misr Dent Sci Res J. (2025) 2:9–19. 10.21608/mdsrj.2025.443694

[9] Al Agili DE. A systematic review of population-based dental caries studies among children in Saudi Arabia. Saudi Dent J. (2013) 25:3–11. 10.1016/j.sdentj.2012.10.002 23960549 PMC3723279

[10] Andegiorgish AK Weldemariam BW Kifle MM Mebrahtu FG Zewde HK Tewelde MG. Prevalence of dental caries and associated factors among 12 years old students in Eritrea. BMC Oral Health. (2017) 17:169. 10.1186/s12903-017-0465-3 29284471 PMC5747091

[11] Nandasena BG Emuck H Paranagama MP Perera K Ratnayake SC Ogawa H. Dental caries experience and associated oral health determinants of 5-6 year old school children in Kandy district, Central Province of Sri Lanka. Open Access J Dent Sci. (2019) 4(4):10-23880.

[12] AlDosari AM Akpata ES Khan N. Associations among dental caries experience, fluorosis, and fluoride exposure from drinking water sources in Saudi Arabia. J Public Health Dent. (2010) 70:220–6. 10.1111/j.1752-7325.2010.00169.x 20459462

[13] Ellakany P Madi M Fouda SM Ibrahim M AlHumaid J. The effect of parental education and socioeconomic status on dental caries among Saudi children. Int J Environ Res Public Health. (2021) 18:11862. 10.3390/ijerph182211862 34831618 PMC8619270

[14] Vasireddy D Sathiyakumar T Mondal S Sur S. Socioeconomic factors associated with the risk and prevalence of dental caries and dental treatment trends in children: a cross-sectional analysis of National Survey of Children's Health (NSCH) data, 2016–2019. Cureus. (2021) 13:e19184. 10.7759/cureus.19184 34873524 PMC8635037

[15] Al-Otaibi M Al-Harthy M Gustafsson A Johansson A Claesson R Angmar-Månsson B. Subgingival plaque microbiota in Saudi Arabians after use of miswak chewing stick and toothbrush. J Clin Periodontol. (2004) 31:1048–53. 10.1111/j.1600-051X.2004.00618.x 15560804

[16] Amin TT Al-Abad BM. Oral hygiene practices, dental knowledge, dietary habits and their relation to caries among male primary school children in Al Hassa, Saudi Arabia. Int J Dent Hyg. (2008) 6:361–70. 10.1111/j.1601-5037.2008.00310.x 19138188

